# In-patient expenditure between 2011 and 2021 for patients with type 2 diabetes mellitus: a hospital-based multicenter retrospective study in southwest China

**DOI:** 10.3389/fpubh.2025.1559424

**Published:** 2025-03-10

**Authors:** Yuxin He, Juntao Tan, Qingzhu Tan, Xiao Zhang, Yunyu Liu, Yang Tang

**Affiliations:** ^1^Department of Medical Administration, Affiliated Banan Hospital of Chongqing Medical University, Chongqing, China; ^2^College of Medical Informatics, Chongqing Medical University, Chongqing, China; ^3^Medical Records and Statistics Room, Affiliated Banan Hospital of Chongqing Medical University, Chongqing, China; ^4^Medical Insurance Department, Affiliated Banan Hospital of Chongqing Medical University, Chongqing, China; ^5^Department of Cardiology, Affiliated Banan Hospital of Chongqing Medical University, Chongqing, China

**Keywords:** type 2 diabetes mellitus, medical expenditure, time trend, multiple linear regression, influencing factors

## Abstract

**Background:**

Type 2 diabetes mellitus (T2DM) is a chronic non-infectious disease that seriously endangers human health. This study aimed to determine the main factors influencing the medical expenditure of T2DM patients and provide guidance for the allocation and control of medical expenditure.

**Methods:**

The homepage data of patients with T2DM were retrospectively collected from six tertiary hospitals in southwest China from January 2011 to December 2021. A multiple linear regression model was constructed to examine the factors associated with medical expenses per patient. Furthermore, the trends of medical expenditure according to other important measures and patient subgroups were described, and a proportional breakdown of medical expenditure was generated. All expenditure data were reported in Chinese Yuan (CNY), based on the 2021 value, and adjusted using the year-specific healthcare consumer price index.

**Results:**

A total of 45,237 patients with T2DM were analyzed in this study. Multiple linear regression showed that age, marital status, insurance type, length of stay, number of clinical visits, number of comorbidities, history of disease, history of surgery, smoking history, and the age-adjusted Charlson comorbidity index score were influencing factors of medical expenditure in patients with T2DM. Considering the overall average medical expenditure, diagnosis cost accounted for the largest proportion and was never <25% since 2011, showing a decreasing trend year by year. Among the types of comorbidity, hypertension was the most prevalent, followed by kidney disease and hyperlipidemia. In terms of the combination of comorbidities, medical expenditure was the highest for pulmonary infection+hypertension (24,360 CNY), followed by coronary heart disease+heart failure+hypertension (22,029 CNY).

**Conclusions:**

Identifying the main factors influencing the medical expenditure of patients with T2DM can provide a reference for the medical security department to formulate reasonable compensation plans and for medical institutions to optimize treatment plans. Ultimately, this might reduce the financial burden of patients and relieve the pressure of medical insurance funds.

## 1 Introduction

The prevalence of diabetes mellitus (DM) is on the rise. This chronic non-infectious disease poses a serious threat to people's health, especially in the context of cardiovascular and cerebrovascular diseases and tumors (1–4). According to the 2019 data from the International Diabetes Federation (IDF), the number of DM patients worldwide rose by 51% (5). In 2019, the number of patients with DM in the world was about 463 million, with China having the highest number of patients (about 116.4 million patients). It is estimated that by 2040, the total number of patients with DM in China will reach 151 million, of which type 2 diabetes mellitus (T2DM) patients will account for more than 90% (6).

In 2019, 10% of the global health expenditure was used for the treatment of DM and its complications. The total medical expenditure for DM is estimated to increase to 825 billion dollars in 2030 and 845 billion dollars in 2045 (7). Furthermore, the growth rate of health expenditure for DM patients in China has already exceeded the growth rate of GDP in the same period (8). The research results of Ding et al. have shown that T2DM accounts for a large proportion of China's medical care expenditure, and this economic burden will continue to increase due to the rising prevalence of DM and its chronic disease characteristics (9). Moreover, the management of DM in China is characterized by a low treatment rate, lack of awareness, and low compliance. DM and its serious complications result in increased direct medical and health costs, labor recession, reduction of effective working hours, etc., which poses a serious economic burden on patients and their families (10–12).

Therefore, it is particularly important to understand the structure of hospitalization expenses of DM patients and its influencing factors. However, few studies have been performed on the medical expenditure of DM at present, and the relevant studies lack representativeness due to the insufficient sample size. Most existing studies focus on small, single-center cohorts, which limits their generalizability and applicability to broader populations. Additionally, previous research has primarily focused on the direct costs of diabetes management, with less attention given to the impact of comorbidities, patient demographics, and healthcare utilization patterns on overall medical expenditure.

This study aims to fill these gaps by analyzing the medical expenditure of 45,237 T2DM patients over a 10-year period (2011–2021) from six tertiary hospitals in southwest China. Unlike previous studies, our research provides a comprehensive analysis of the factors influencing medical expenditure, including age, marital status, insurance type, length of stay (LOS), number of clinical visits, comorbidities, and the age-adjusted Charlson comorbidity index (ACCI) score. By focusing on a large, diverse patient population over an extended period, this study offers valuable insights into the long-term trends and determinants of medical expenditure for T2DM patients, which can inform healthcare policy and resource allocation strategies.

## 2 Methods

### 2.1 Study design and patients

In this study, the clinical data of 62,741 patients with T2DM were collected from six tertiary general hospitals in southwest China, of which 45,237 patients passed quality control for the final analysis. These hospitals were selected because they represent high-level medical institutions with comprehensive clinical, teaching, and research capabilities, which are essential for addressing the objectives of this study. Tertiary general hospitals in China typically serve as regional healthcare hubs, providing advanced medical services and handling complex cases. The inclusion of these hospitals ensures a consistent level of healthcare quality and resource availability, which is critical for the generalizability of the findings within similar healthcare settings. The study included data on age, sex, marital status, insurance type, LOS, number of clinical visits, number of comorbidities, history of disease, history of surgery, smoking history, drinking history, and the ACCI score. This study was approved by the Ethics Committee of the Affiliated Banan Hospital of Chongqing Medical University (Ethical approval No. BNLLKY2023037). Informed consent for participation was not required for this study owing to its retrospective design, and the study was conducted in accordance with national legislation and institutional requirements.

### 2.2 Inclusion and exclusion criteria

The inclusion criteria were: (i) data obtained from January 2011 to December 2021, and (ii) hospitalizations for T2DM. The exclusion criteria were: (i) patients with missing medical expenditure; (ii) hospitalization days <1 or >60; (iii) age <18 years; and, (iv) patients with extremely low (<1st percentile) or high (>99th percentile) medical expenditures. The selection process is illustrated in Supplementary Figure S1.

Patients with a LOS of <1 day were excluded because such short stays often represent outpatient visits or incomplete hospitalization records, which do not align with the study's focus on inpatient care. On the other hand, patients with LOS exceeding 60 days were excluded because such prolonged stays are typically associated with severe complications, non-routine treatments, or exceptional clinical circumstances that are not representative of the average T2DM patient's hospitalization experience. Including these extreme cases could introduce bias and distort the analysis of typical medical expenditures for T2DM patients. By excluding these outliers, we ensure that the study results reflect the general trends and factors influencing medical expenditure for the majority of T2DM inpatients. Patients with medical expenditures falling outside the 1st and 99th percentiles were excluded from the analysis to minimize the influence of outliers and ensure data consistency. This approach was adopted to focus on typical cost patterns and enhance the clinical relevance of our findings.

### 2.3 Definition

The main outcome measure of this study was medical expenditure per patient. Medical expenditures included consumable costs, drug costs, treatment costs, diagnosis costs, comprehensive medical service charges, and other costs. The insurance type reflects the economic burden borne by individuals and families. In this study, the insurance type was divided into the following five categories: urban employee medical insurance (UEMI), new cooperative medical scheme (NCMS), urban resident medical insurance (URMI), other medical insurance, and fully self-paid. The number of comorbidities was defined as the total number of comorbidities affecting patients out of the top 11 most frequent comorbidities observed in the study population. These comorbidities included hyperlipidemia, kidney disease, coronary heart disease (CHD), cancer, chronic obstructive pulmonary disease (COPD), atrial fibrillation (AF), cerebral infarction (CI), pulmonary infection, osteoporosis, heart failure (HF), and hypertension. Importantly, T2DM itself was not counted as a comorbidity in this definition. The ACCI score, however, was calculated based on the Charlson Comorbidity Index, which includes T2DM as a baseline condition with a score of 1 for uncomplicated diabetes and an additional score of 2 for end-organ damage.

### 2.4 Statistical analyses

Statistical analyses were performed using SPSS 22.0 and R (version 4.3.2, Vienna, Austria). Due to the long duration of this study, medical expenditure data were reported in Chinese Yuan (CNY) based on the 2021 value, which was adjusted using the year-specific personal healthcare consumer price index (CPI) of Chongqing. Univariate analyses were carried out to determine the significance of observed differences in medical expenditure using a two-sample student's *t*-test or an ANOVA test after the logarithm transition. Subsequently, a multiple linear regression model was constructed to determine the factors associated with medical expenses per patient. To ensure the validity of the regression model, we conducted diagnostic tests to assess key regression assumptions. First, multicollinearity was evaluated using the variance inflation factor (VIF), with a threshold of VIF < 10 indicating no significant multicollinearity among the independent variables. Second, the normality of residuals was assessed using the Shapiro-Wilk test and visual inspection of Q-Q plots. Third, heteroscedasticity was examined using the Breusch-Pagan test, and no evidence of heteroscedasticity was found. These tests confirmed that the regression model met the necessary assumptions, ensuring the robustness and reliability of the results. Furthermore, the trends of medical expenditure based on other important measures and patient subgroups were described, and a proportional breakdown of medical expenditure was generated. Moreover, the status of medical expenditure in different combinations of comorbidities was analyzed. All statistical analyses were two-sided, and statistical significance was set at *P* < 0.05.

## 3 Results

### 3.1 Patient characteristics

A total of 45,237 patients suffering from T2DM were enrolled, including 24,980 females (55.22%) and 20,257 males (44.78%); 40.23% were ≥65 years old; the median LOS was 8 days (P25–P75: 5–12) and the majority of the patients (91.28%) were married. A large number of patients had medical insurance coverage (81.09%), with UEMI accounting for the largest proportion (40.62%). Patients with a history of disease or history of surgery constituted 81.03 and 45.02% of the participants, respectively (Table 1). Supplementary Table S1 displays the socio-demographic and clinical-pathological characteristics of the 45,237 selected patients between 2011 and 2021.

**Table 1:** Demographic and clinical characteristics of 45,237 T2DM patients (2011–2021).

**Variables**	**Number of patients [cases (%)]**
**Age (years)**	
18–44	9,594 (21.21)
45–64	17,442 (38.56)
65–84	16,987 (37.55)
≥85	1,214 (2.68)
**Sex**	
Female	24,980 (55.22)
Male	20,257 (44.78)
**Marital status**	
Unmarried	1,011 (2.23)
Married	41,291 (91.28)
Divorced/widowed	2,935 (6.49)
**Insurance type**	
Full self-pay	8,554 (18.91)
UEMI	18,375 (40.62)
URMI	14,458 (31.96)
NCMS	451 (1.00)
Other medical insurance	3,399 (7.51)
**Length of stay (days)**	
2–4	8,302 (18.35)
5–7	13,105 (28.97)
8–11	12,164 (26.89)
≥12	11,666 (25.79)
**Number of clinical visits**	
1	26,621 (58.85)
2	8,981 (19.85)
3	3,917 (8.66)
≥4	5,718 (12.64)
**Number of comorbidities** ^a^	
0	23,414 (51.76)
1	8,958 (19.81)
2	6,769 (14.96)
3	3,715 (8.21)
≥4	2,381 (5.26)
**History of disease**	
No	8,580 (18.97)
Yes	36,657 (81.03)
**History of surgery**	
No	24,870 (54.98)
Yes	20,367 (45.02)
**Smoking history**	
No	32,376 (71.57)
Yes	12,861 (28.43)
**Drinking history**	
No	33,995 (75.15)
Yes	11,242 (24.85)
**ACCI score** ^b^	
0–1	7,553 (16.70)
2–3	10,546 (23.31)
4–5	13,831 (30.57)
≥6	13,307 (29.42)

UEMI, urban employee medical insurance; URMI, urban resident medical insurance; NCMS, new cooperative medical scheme; ACCI, age-adjusted charlson comorbidity index.

^a^The number of comorbidities was defined based on the top 11 most frequent comorbidities observed in the study population, excluding T2DM.

^b^The ACCI score was calculated based on the Charlson Comorbidity Index, which includes T2DM as a baseline condition with a score of 1 for uncomplicated diabetes and an additional score of 2 for end-organ damage.

### 3.2 Analysis of influencing factors of medical expenditure

The univariate analysis revealed that expenditure differed according to age, sex, marital status, insurance type, LOS, number of clinical visits, number of comorbidities, history of disease, history of surgery, smoking history, drinking history, and the ACCI score (*P* < 0.05). However, more recent data could better reflect the current and future situation, so the expenditure data for the final 3 years (2019–2021) was further analyzed (Table 2). The average expenditure of most subgroups between 2019 and 2021 was higher than that between 2011 and 2021. Furthermore, multiple linear regression revealed that age, marital status, insurance type, LOS, number of clinical visits, number of comorbidities, history of disease, history of surgery, smoking history, and the ACCI score were factors influencing medical expenditure in patients with T2DM (Table 3).

**Table 2:** Univariate analysis of factors associated with medical expenditure in T2DM patients.

**Variables**	**Expenditure per patient during 2011–2021 (CNY)**	**Statistics**	***P*-value**	**Expenditure per patient during 2019–2021 (CNY)**	**Statistics**	***P*-value**
Overall	12,335 (12,219–12,451)			13,769 (13,574–13,964)		
Age (years)		473.874	< 0.001^a^		204.656	< 0.001^a^
18–44	8,879 (8,735–9,023)			9,678 (9,434–9,922)		
45–64	12,126 (11,939–12,313)			13,719 (13,399–14,039)		
65–84	14,300 (14,085–14,515)			15,927 (15,571–16,283)		
≥85	15,157 (14,358–15,956)			15,870 (14,687–17,062)		
Sex		160.757	< 0.001^b^		115.763	< 0.001^b^
Female	11,426 (11,286–11,566)			12,798 (12,558–13,038)		
Male	13,456 (13,264–13,648)			14,890 (14,576–15,204)		
Marital status		49.014	< 0.001^a^		4.842	0.008^a^
Unmarried	11,254 (10,526–11,982)			12,766 (11,492–14,040)		
Married	12,417 (12,297–12,537)			13,785 (13,581–13,989)		
Divorced/ widowed	11,561 (11,101–12,021)			13,887 (13,116–14,658)		
Insurance type		460.031	< 0.001^a^		241.437	< 0.001^a^
Full self-pay	9,343 (9,123–9,563)			9,804 (9,450–10,158)		
UEMI	13,326 (13,133–13,519)			14,516 (14,209–14,823)		
URMI	13,403 (13,188–13,618)			14,863 (14,530–15,196)		
NCMS	12,849 (11,756–13,942)			14,549 (12,446–16,652)		
Other medical insurance	9,897 (9,592–10,202)			13,464 (11,925–15,003)		
Length of stay (days)		8582.217	< 0.001^a^		3754.358	< 0.001^a^
2–4	5,881 (5,788–5,974)			6,521 (6,365–6,677)		
5–7	8,244 (8,133–8,355)			9,012 (8,831–9,193)		
8–11	11,758 (11,577–11,939)			13,186 (12,872–13,500)		
≥12	22,126 (21,810–22,442)			25,549 (25,011–26,087)		
Number of clinical visits		31.970	< 0.001^a^		11.803	< 0.001^a^
1	12,067 (11,918–12,216)			13,400 (13,167–13,633)		
2	12,320 (12,058–12,582)			13,956 (13,503–14,409)		
3	12,361 (11,976–12,746)			13,824 (13,114–14,534)		
≥4	13,590 (13,240–13,940)			16,116 (15,319–16,913)		
Number of comorbidities^c^		625.709	< 0.001^a^		463.683	< 0.001^a^
0	9,935 (9,825–10,045)			10,083 (9,887–10,279)		
1	13,127 (12,836–13,418)			14,564 (14,134–14,994)		
2	14,609 (14,249–14,969)			16,140 (15,617–16,663)		
3	17,014 (16,475–17,553)			18,867 (18,084–19,650)		
≥4	19,198 (18,493–19,903)			20,982 (19,994–21,970)		
History of disease		238.030	< 0.001^b^		171.740	< 0.001^b^
No	9,956 (9,747–10,165)			10,801 (10,480–11,122)		
Yes	12,892 (12,758–13,026)			14,537 (14,308–14,766)		
History of surgery		23.104	< 0.001^b^		14.281	< 0.001^b^
No	11,804 (11,655–11,953)			13,101 (12,854–13,348)		
Yes	12,984 (12,802–13,166)			14,610 (14,300–14,920)		
Smoking history		98.346	< 0.001^b^		49.654	< 0.001^b^
No	11,631 (11,503–11,759)			12,963 (12,746–13,180)		
Yes	14,109 (13,860–14,358)			15,663 (15,260–16,066)		
Drinking history		32.728	< 0.001^b^		10.514	0.001^b^
No	11,927 (11,798–12,056)			13,370 (13,151–13,589)		
Yes	13,571 (13,317–13,825)			14,925 (14,511–15,339)		
ACCI score^d^		508.783	< 0.001^a^		280.729	< 0.001^a^
0–1	8,423 (8,314–8,532)			9,088 (8,910–9,266)		
2–3	10,849 (10,649–11,049)			11,738 (11,376–12,100)		
4–5	12,988 (12,765–13,211)			14,400 (14,017–14,783)		
≥6	15,055 (14,795–15,315)			16,731 (16,339–17,123)		

UEMI, urban employee medical insurance; URMI, urban resident medical insurance; NCMS, new cooperative medical scheme; ACCI, age-adjusted charlson comorbidity index; CNY, Chinese Yuan.

All expenditure data were presented as mean with 95% confidence interval in parentheses.

^a^ANOVA test after logarithm transition.

^b^Two-sample Student t-test after logarithm transition.

^c^The number of comorbidities was defined based on the top eleven most frequent comorbidities observed in the study population, excluding T2DM.

^d^The ACCI score was calculated based on the Charlson Comorbidity Index, which includes T2DM as a baseline condition with a score of 1 for uncomplicated diabetes and an additional score of 2 for end-organ damage.

**Table 3:** Multivariate analysis of factors associated with medical expenditure in T2DM patients.

**Variables**	**Analysis 1: medical expenditure during 2011–2021** ^**a**^					**Analysis 2: medical expenditure during 2019–2021** ^**a**^				
	**Conffificient**	**SE**	**95%CI**		***P*** **-value**	**Conffificient**	**SE**	**95%CI**		***P*** **-value**
			**Lower**	**Upper**				**Lower**	**Upper**	
**Age (years)**										
18–44	Reference					Reference				
45–64	−0.004	0.004	−0.013	0.004	0.341	−0.009	0.007	−0.023	0.005	0.192
65–84	0.032	0.005	0.023	0.042	< 0.001	0.02	0.008	0.005	0.035	0.009
≥85	0.059	0.009	0.042	0.075	< 0.001	0.031	0.013	0.005	0.056	0.019
**Sex**										
Female	/	/	/	/	/	/	/	/	/	/
Male	/	/	/	/	/	/	/	/	/	/
**Marital status**										
Unmarried	Reference					Reference				
Married	0.035	0.008	0.019	0.050	< 0.001	0.033	0.012	0.009	0.057	0.007
Divorced/widowed	−0.030	0.009	−0.048	−0.013	0.001	0.006	0.014	−0.021	0.034	0.646
**Insurance type**										
Full self-pay	Reference					Reference				
UEMI	0.077	0.003	0.071	0.084	< 0.001	0.094	0.005	0.084	0.104	< 0.001
URMI	0.113	0.003	0.106	0.119	< 0.001	0.124	0.005	0.114	0.135	< 0.001
NCMS	0.087	0.012	0.064	0.109	< 0.001	0.145	0.024	0.099	0.192	< 0.001
Other medical insurance	−0.005	0.005	−0.015	0.005	0.320	0.096	0.018	0.060	0.132	< 0.001
**Length of stay (days)**										
2–4	Reference					Reference				
5–7	0.142	0.003	0.136	0.149	< 0.001	0.132	0.005	0.122	0.143	< 0.001
8–11	0.281	0.004	0.274	0.288	< 0.001	0.269	0.005	0.258	0.28	< 0.001
≥12	0.535	0.004	0.528	0.542	< 0.001	0.538	0.006	0.527	0.549	< 0.001
**Number of clinical visits**										
1	Reference					Reference				
2	−0.026	0.003	−0.032	−0.02	< 0.001	−0.018	0.005	−0.027	−0.009	< 0.001
3	−0.038	0.004	−0.047	−0.03	< 0.001	−0.027	0.007	−0.041	−0.014	< 0.001
≥4	−0.044	0.004	−0.051	−0.036	< 0.001	−0.024	0.007	−0.037	−0.011	< 0.001
**Number of comorbidities** ^b^										
0	Reference					Reference				
1	0.037	0.003	0.031	0.044	< 0.001	0.07	0.005	0.06	0.08	< 0.001
2	0.064	0.004	0.057	0.072	< 0.001	0.096	0.006	0.085	0.108	< 0.001
3	0.117	0.005	0.108	0.126	< 0.001	0.15	0.007	0.136	0.164	< 0.001
≥4	0.163	0.006	0.152	0.175	< 0.001	0.195	0.008	0.178	0.211	< 0.001
**History of disease**										
No	Reference					Reference				
Yes	0.022	0.003	0.016	0.029	< 0.001	0.028	0.005	0.019	0.038	< 0.001
**History of surgery**										
No	Reference					/	/	/	/	/
Yes	0.006	0.003	0.001	0.011	0.019	/	/	/	/	/
**Smoking history**										
No	Reference					Reference				
Yes	0.024	0.003	0.019	0.029	< 0.001	0.024	0.004	0.017	0.032	< 0.001
**Drinking history**										
No	/	/	/	/	/	/	/	/	/	/
Yes	/	/	/	/	/	/	/	/	/	/
**ACCI score** ^c^										
0–1	Reference					Reference				
2–3	−0.087	0.005	−0.096	−0.077	< 0.001	−0.114	0.008	−0.13	−0.099	< 0.001
4–5	−0.076	0.005	−0.087	−0.066	< 0.001	−0.1	0.008	−0.117	−0.084	< 0.001
≥6	−0.090	0.006	−0.101	−0.078	< 0.001	−0.116	0.009	−0.134	−0.098	< 0.001
Adjusted *R*^2^	0.422	/	/	/	/	0.414	/	/	/	/

UEMI, urban employee medical insurance; URMI, urban resident medical insurance; NCMS, new cooperative medical scheme; ACCI, age-adjusted charlson comorbidity index; SE, standard error; CI, confidence interval.

^*a*^The logarithmic transformation of the medical expenditure data was performed in the multivariate regression analysis.

^*b*^The number of comorbidities was defined based on the top 11 most frequent comorbidities observed in the study population, excluding T2DM.

^*C*^The ACCI score was calculated based on the Charlson Comorbidity Index, which includes T2DM as a baseline condition with a score of 1 for uncomplicated diabetes and an additional score of 2 for end-organ damage.

### 3.3 Time trends of medical expenditure and related factors

**Figure 1:**
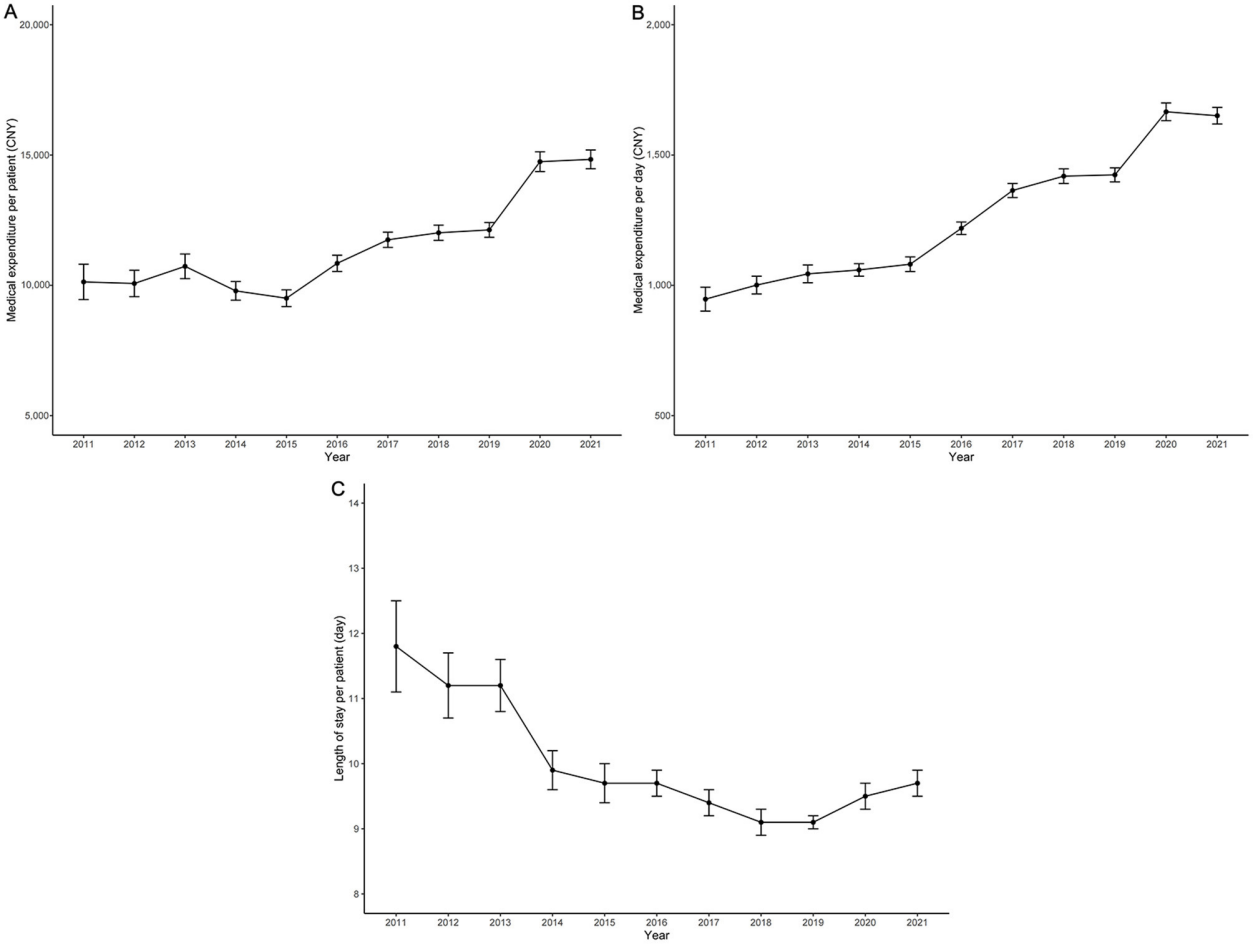
Trends in medical expenditure and related factors for T2DM patients (2011–2021). **(A)** Trends in overall average medical expenditure per patient; **(B)** Trends in daily average medical expenditure; **(C)** Trends in length of stay per patient. The y-axis represents costs in CNY or days, and the x-axis represents the years.

Figure 1 shows medical expenditure time trends and related factors for patients with T2DM over the 2011–2021 period. The overall average expenditure per patient increased by 3.89% per year from 10,132 CNY in 2011 to 14,834 CNY in 2021 (Figure 1A). In addition, the daily average expenditure increased by 5.72% per year from 947 CNY in 2011 to 1,651 CNY in 2021 (Figure 1B). A turning point was observed around 2019, with the LOS per patient decreasing by 3.21% per year before 2019, and then slightly increasing between 2019 and 2021 (Figure 1C).

### 3.4 Medical expenditure time trends according to patient subgroups (2011–2021)

**Figure 2:**
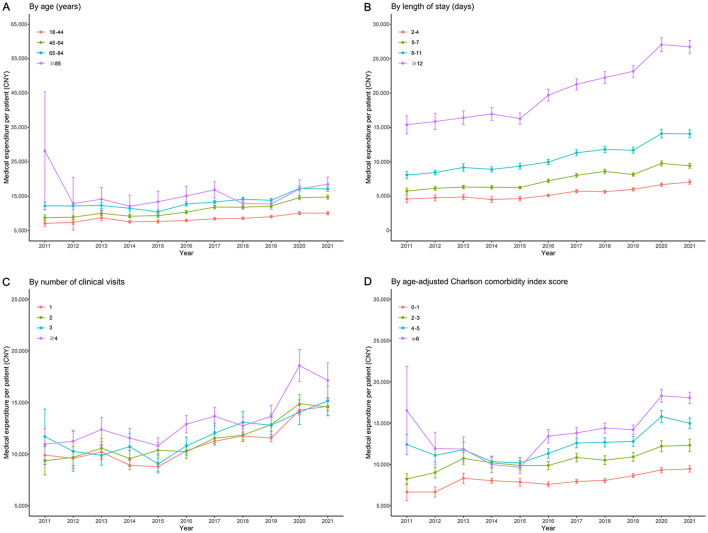
Subgroup analysis of medical expenditure trends in T2DM patients (2011–2021). **(A)** Trends stratified by age groups; **(B)** Trends stratified by LOS; **(C)** Trends stratified by the number of clinical visits; **(D)** Trends stratified by the Age-Adjusted Charlson Comorbidity Index (ACCI) score. The y-axis represents costs in CNY, and the x-axis represents the years.

The time trend of average expenditures differed according to age, LOS, number of clinical visits, and the ACCI score (Figure 2). The average medical expenditure per patient aged ≥85 years was higher than that for patients aged 18–44 years, 45–64 years, and 65–84 years (Figure 2A). Additionally, the average medical expenditure per patient with LOS ≥12 days was considerably higher than that for LOS 2–4 days, 5–7 days, and 8–11 days, and the gap between LOS ≥12 days and other LOS subgroups gradually increased (Figure 2B). Overall, the average medical expenditure per patient with ≥4 clinical visits was higher than those with 1, 2, and 3 clinical visits. In contrast, no significant difference in average medical expenditure per patient was observed between patients with 1, 2, and 3 clinical visits (Figure 2C). The average medical expenditure per patient for those with ACCI score ≥6 was much higher than those with ACCI scores 0–1, 2–3, and 4–5, and the gap increased after 2015 (Figure 2D). Further details are also presented according to the history of disease, history of surgery, and smoking history (Supplementary Figures S2–S4).

### 3.5 The proportional breakdown of medical expenditure (2011–2021)

**Figure 3:**
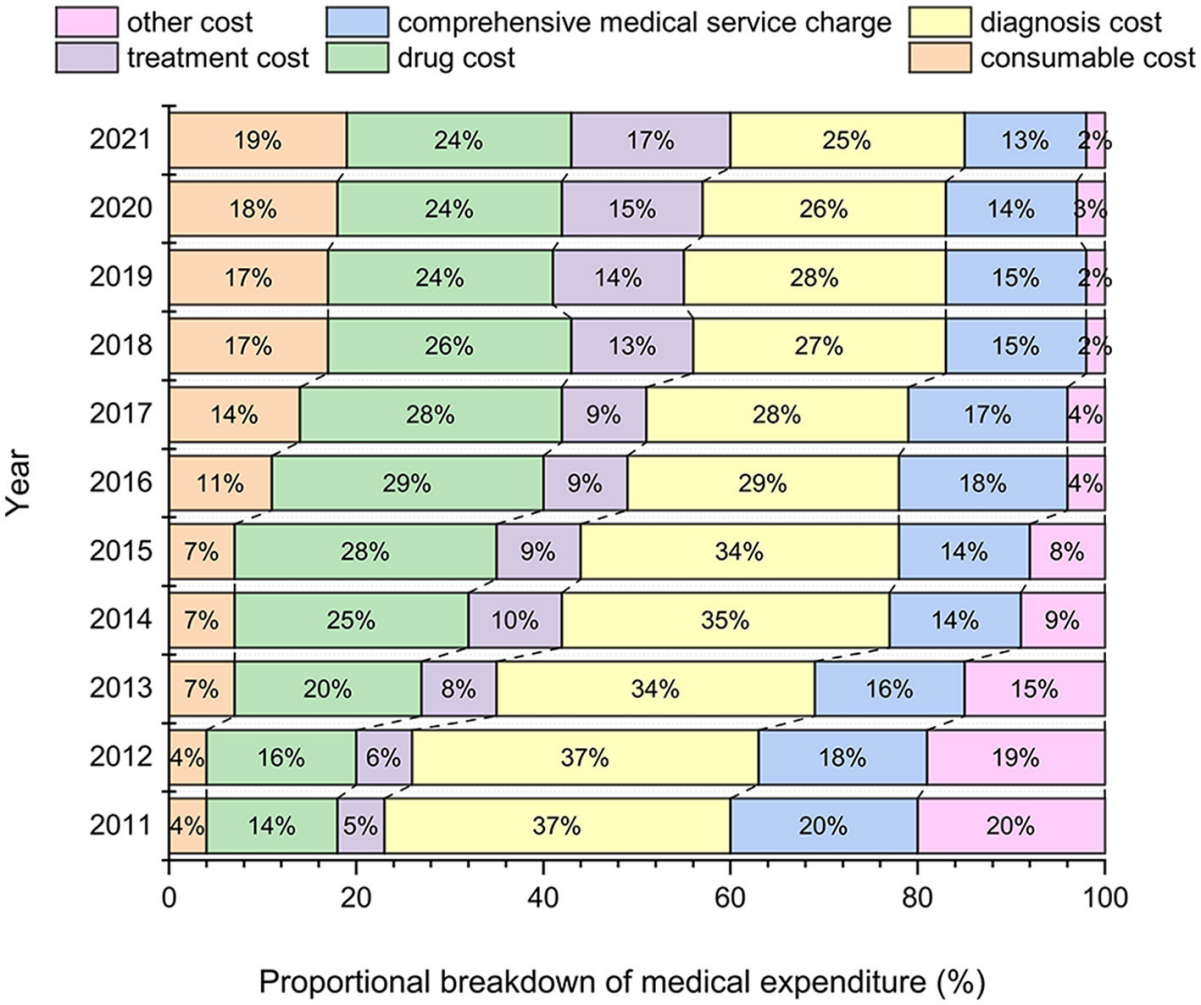
Proportional breakdown of medical expenditure for T2DM diagnosis and treatment.

Diagnosis cost accounted for the largest proportion of the overall average medical expenditure and was never < 25% since the year 2011, but showed a decreasing trend year by year (Figure 3). In contrast, the proportion of consumable cost and treatment cost dramatically increased from 4.00% in 2011 to 19.00% in 2021, and 5.00% in 2011 to 17.00% in 2021, respectively. A turning point was observed around 2016, with the proportion of drug costs increasing from 14.00% in 2011 to 29.00% in 2016, and then slightly decreasing between 2016 and 2021.

### 3.6 Medical expenditure in different types of comorbidities

Hypertension (*n* = 14,450) was the most prevalent comorbidity co-occurring with T2DM, followed by kidney disease (*n* = 6,683) and hyperlipidemia (*n* = 5,090). However, the medical expenditure of T2DM comorbid with pulmonary infection was the highest (23,446 CNY), followed by HF (19,646 CNY), AF (19,631 CNY), CHD (19,454 CNY), and cancer (19,093 CNY; Table 4).

**Table 4:** Medical expenditure by comorbidity type in T2DM patients.

**Type of comorbidity**	***n***	**Consumable cost**	**Drug cost**	**Treatment cost**	**Diagnosis cost**	**Comprehensive medical service charge**	**Other cost**	**Medical expenditure**	**Rank**
Pulmonary infection	1,802	3,148	8,593	1,812	5,598	4,015	280	23,446	1
HF	2,257	4,859	5,752	1,696	4,508	2,721	110	19,646	2
AF	932	3,459	6,177	1,980	4,564	3,162	289	19,631	3
CHD	4,895	5,355	5,137	2,274	4,208	2,398	82	19,454	4
Cancer	2,072	2,929	6,114	2,005	4,750	3,163	132	19,093	5
COPD	1,096	2,661	6,225	1,359	4,597	3,207	114	18,163	6
CI	3,351	1,879	5,642	1,689	4,255	2,695	348	16,508	7
Hypertension	14,450	3,077	4,774	1,935	3,842	2,353	148	16,129	8
Kidney disease	6,683	2,493	4,581	1,411	4,425	2,324	146	15,380	9
Osteoporosis	1,527	2,728	3,879	1,948	4,182	2,327	262	15,326	10
Hyperlipidemia	5,090	2,572	4,348	1,272	3,886	1,910	92	14,080	11

HF, heart failure; AF, atrial fibrillation; CHD, coronary heart disease; COPD, chronic obstructive pulmonary disease; CI, cerebral infarction.

### 3.7 Medical expenditure in different combinations of comorbidities

**Figure 4:**
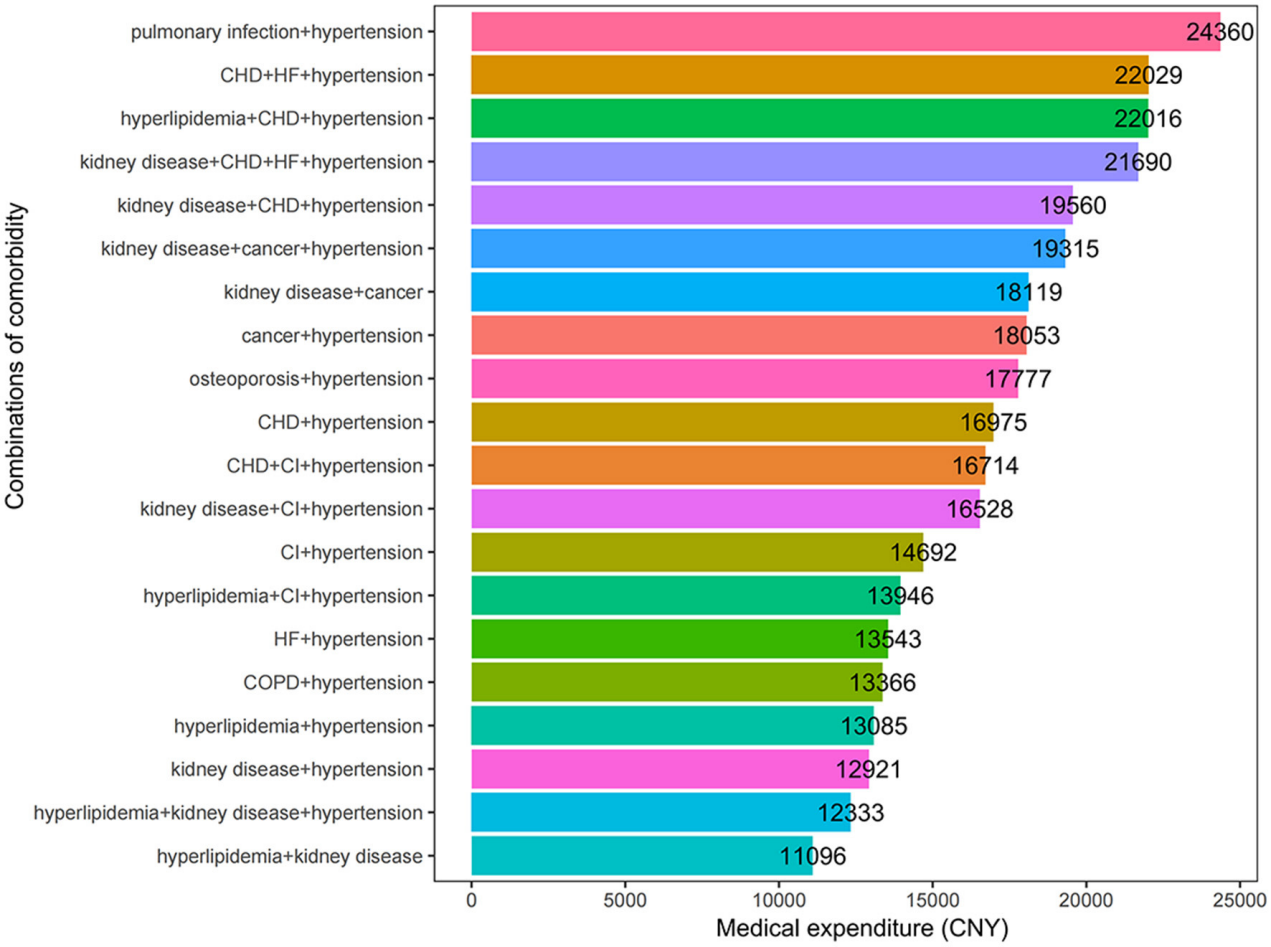
Medical expenditure for different comorbidity combinations in T2DM patients.

The 20 comorbidities most frequently accompanying T2DM are listed in Figure 4. The five comorbidities with the highest average medical expenditure were (1) pulmonary infection+hypertension (24,360 CNY), (2) CHD+HF+hypertension (22,029 CNY), (3) hyperlipidemia+CHD+hypertension (22,016 CNY), (4) kidney disease+CHD+HF+hypertension (21,690 CNY), and (5) kidney disease+CHD+hypertension (19,560 CNY). Supplementary Table S2 provides a detailed overview of the patient distribution across various comorbidity combinations, accompanied by their respective medical expenditures.

## 4 Discussion

T2DM is an incurable disease and generally requires lifelong care and medication. In addition, advances in treatment technology and the higher prevalence of T2DM in young individuals impose a large economic burden on patients and society. Medical expenditure directly reflects T2DM consumption, which enables the monitoring, prevention, and control of the disease. Our study provided a detailed description of the influencing factors of medical expenditure for T2DM patients in China. The medical expenditure of T2DM patients was related to the following factors: age, marital status, insurance type, LOS, number of clinical visits, number of comorbidities, history of disease, history of surgery, smoking history, and the ACCI score. Among the types of comorbidities, hypertension, and kidney disease showed the highest co-occurrence with T2DM.

In this study, we observed that 51.76% of patients had no comorbidities other than T2DM, while 83.30% had an ACCI score ≥2. This apparent discrepancy can be explained by the fact that the number of comorbidities was defined based on the top 11 most frequent comorbidities, excluding T2DM itself. In contrast, the ACCI score was calculated based on the Charlson Comorbidity Index, which includes T2DM as a baseline condition with a score of 1 for uncomplicated diabetes and an additional score of 2 for end-organ damage. Therefore, even patients with no additional comorbidities would have an ACCI score of at least 1, while those with additional comorbidities or end-organ damage would have higher scores. This highlights the importance of clearly defining comorbidity counts and their relationship to comorbidity indices such as the ACCI score.

In 2014, the cost associated with DM in China was 80.33 billion CNY, accounting for nearly 5% of chronic disease medical resources, and 5.4% of social security funds for chronic disease medical expense compensation. In 2020, the treatment cost of DM reached 143.74 billion CNY, with about 33.4% being funded by patients themselves (13). From the perspective of cost structure, the increase in hospitalization costs for patients with DM has a significant impact on the medical fund pool (14).

Previous studies have reported that the most direct factor affecting the medical expenditure of patients was the LOS (14–17), which is consistent with the results of our study. Therefore, reducing the LOS of patients is an important means of cost control. Clinicians should be encouraged to adopt standardized treatment paths on the premise of ensuring medical quality and formulate diagnosis and treatment plans according to different patients' conditions. Moreover, long-term hospital stays should be avoided by transferring the improved patients to the community for follow-up rehabilitation treatment, resulting in a higher bed turnover rate. Shortening the hospitalization time of patients not only reduces the cost burden of patients but also improves the medical efficiency of the hospital.

The present study revealed that comorbidities significantly affected the medical expenditure of T2DM patients. Comorbidities have been proven to have adverse effects on the pharmacological management (18) and flash continuous glucose monitoring (19) of T2DM patients, leading to prolonged LOS and increased risk of postoperative complications (20). In addition, a higher number of comorbidities was associated with higher medical expenses for T2DM patients. The research results of Chi et al. demonstrated that the superposition, coexistence, and combination of multiple chronic diseases would result in the overconsumption of medical resources, thus increasing medical expenditure (21). Relevant research has also shown a 1.73 increase in hospital stay and a 1.34 increase in medical for each additional chronic disease comorbidity (22). Therefore, limiting the number of comorbidities in T2DM patients is crucial to reducing medical expenditure. Among the common comorbidities related to T2DM, pulmonary infection involves the highest medical expenditure, followed by HF. In terms of combinations of comorbidities, pulmonary infection+hypertension was associated with the highest medical expenditure. Therefore, pulmonary infection cannot be ignored in the context of T2DM.

The medical expenditure of T2DM patients with insurance types NCMS, UEMI, and URMI were significantly higher than that of T2DM patients with other medical insurance and fully self-paid patients. As the most mature medical insurance project, UEMI can be traced back to the mid-1990s (23). China has piloted the NCMS insurance model in rural areas since 2003 and basically achieved full coverage in rural areas in 2010 (24). In order to fill the coverage gap in the existing medical system and provide medical insurance for urban non-employees, the Chinese government piloted the URMI model in 79 cities in 2007 and promoted it nationwide in 2009 (25). The coverage of NCMS increased rapidly from 11.63% in 2005 to 80.34% in 2014. The medical expenditure of the older adults also increased from an average of $204.77 in 2005 to $696.23 in 2014 (26). Moreover, the pooling fund per capita for URMI increased from $20.44 in 2008 to $79.31 in 2015. At the same time, the number of insured persons in URMI increased from 118.26 million in 2008 to 376.89 million in 2015 (27).

The higher medical expenditure observed among insured patients may be attributed to both broader insurance coverage and increased utilization of medical services. Insured patients are more likely to seek medical care due to reduced out-of-pocket costs, leading to higher utilization of diagnostic tests, treatments, and hospitalizations. Additionally, insured patients may receive more comprehensive care, including the management of comorbidities and complications, which can further drive up costs. On the other hand, uninsured or self-paid patients may delay seeking care or opt for less expensive treatment options, resulting in lower overall medical expenditure. Therefore, the higher costs observed among insured patients likely reflect both the broader coverage provided by insurance and the increased utilization of medical services. With the aging population and the demand for higher-quality medical services, NCMS, UEMI, and URMI are facing huge financial payment pressure. Therefore, it is particularly important to strengthen the function of interest integration, improve the medical insurance system, and give full play to the main guarantee function of basic medical insurance.

A weak correlation was found between the age of patients and medical expenditure. The comparison results of the age groups in Table 2 show that there is no significant difference in the medical expenditure of T2DM patients under 65 years old, while there is a difference in the medical expenditure of patients above this age group. The epidemiology, clinical characteristics, course of disease, treatment, and monitoring strategies of T2DM patients vary with the age of onset (28). This may lead to the heterogeneity of T2DM medical expenditure, highlighting the need for further research on the medical expenditure of T2DM patients of different ages. Furthermore, previous studies have shown that marital status, number of clinical visits, history of disease, history of surgery, smoking history, and the ACCI score exerted different effects on the medical expenditure of in-patients, which is consistent with the results of our study (29–32).

This study also compared the time trend of medical expenditure for T2DM in different subgroups. Patients aged ≥ 85 years showed the highest average medical expenditure, which may be attributed to the fact that diabetes in older adults is associated with higher mortality, lower functional status, and higher risk of long-term complications (33). The average medical expenditure per patient increased with LOS, the number of clinical visits, and the ACCI score. In addition, the average medical expenditure of patients with a history of disease was higher than that of patients without history of disease and was higher in patients with a history of surgery compared to those without. Meanwhile, a higher average medical expenditure was observed with patients with a smoking history compared to patients without a smoking history. Compared with people who never smoked, a significant increase in the risk of adverse health outcomes caused by smoking was observed in current and former smokers (34). A study showed that more than 50% of annual healthcare expenditure can be attributed to smoking (35).

The observed increase in medical costs after 2019 may be attributed to several factors, including policy changes and external events. In 2019, China implemented the National Centralized Drug Procurement (NCDP) policy, also known as the “4 + 7” policy, which aimed to reduce drug prices by centralizing the procurement of certain medications (36). While this policy initially led to a reduction in drug costs, it also resulted in increased costs for other medical services, such as diagnostics and treatments, as hospitals sought to compensate for reduced revenue from drug sales. Additionally, the Diagnosis-Related Group (DRG) payment reform was piloted in Chongqing in 2018, which standardized reimbursement rates based on disease severity and treatment complexity. This reform may have contributed to the stabilization of medical expenditure growth after 2019, as hospitals were incentivized to control costs while maintaining quality of care. Another important policy was the Healthy China 2030 Plan, launched in 2016, which emphasized the prevention and management of chronic diseases, including diabetes (37). This plan likely increased awareness and early diagnosis of T2DM, leading to higher hospitalization rates and associated costs. Furthermore, the expansion of reimbursement for diabetes-related medications and treatments under the NCMS and URMI during this period may have contributed to the observed increase in medical expenditure, particularly for patients with comorbidities such as hypertension and kidney disease. Additionally, the COVID-19 pandemic, which began in late 2019, significantly impacted healthcare systems worldwide, including in China (38). The pandemic led to increased costs for infection control measures, personal protective equipment, and additional healthcare resources, which may have contributed to the rise in medical expenditures observed in this study. Furthermore, the pandemic disrupted routine healthcare services, leading to delayed treatments and more severe complications for chronic disease patients, including those with T2DM, which could have further increased costs. Future studies should explicitly incorporate policy variables to better understand their impact on healthcare costs.

Our findings align with several international studies on medical expenditure for T2DM patients, though some differences exist due to variations in healthcare systems and patient populations. For instance, a study in the United States found that longer hospital stays and comorbidities, such as hypertension and cardiovascular diseases, significantly increased medical costs for T2DM patients, consistent with our results (39). Similarly, research in European countries, such as Germany and the UK, highlighted that integrated care models and early comorbidity management effectively reduced costs, supporting our recommendation for prioritizing comorbidity screening and management (40, 41). However, differences in insurance systems and healthcare delivery models also lead to variations in cost trends. For example, in countries with universal healthcare systems, such as Canada and Australia, the proportion of out-of-pocket expenses for T2DM patients is lower compared to our findings, where insurance type significantly influences expenditure (42, 43). Additionally, studies from low- and middle-income countries, such as India and Brazil, emphasize the role of limited access to advanced treatments and diagnostics in driving costs, which contrasts with our observation of rising consumable and treatment costs due to technological advancements (44, 45).

This study highlights key factors driving medical expenditure for T2DM patients and offers actionable recommendations for health policy. Optimizing length of stay through standardized protocols and community-based care can reduce costs and improve hospital efficiency. Prioritizing early detection and management of comorbidities, such as pulmonary infections and hypertension, can prevent complications and lower expenses. Reforming insurance reimbursement mechanisms to incentivize cost-effective care, alongside investing in preventive care and patient education, can reduce clinical visits and complications. Additionally, adopting cost-effective technologies like telemedicine can lower treatment costs while maintaining care quality. These strategies collectively support cost-effective healthcare delivery without compromising outcomes.

Despite the comprehensive analysis of medical expenditures and comorbidities in this study, several limitations should be acknowledged. First, our analysis was limited to tertiary hospitals, which may underrepresent patients treated in primary clinics or secondary hospitals. While this focus was appropriate for addressing the study's objectives, future research could expand data coverage to include a broader range of healthcare facilities to provide a more representative picture of the healthcare system. Second, although all included hospitals are affiliated with Chongqing Medical University and follow standardized protocols for cost accounting and diagnosis coding, minor variations in practices may still exist. To address this, we conducted rigorous data cleaning and validation processes and normalized medical expenditure data using the year-specific healthcare consumer price index (CPI). These steps helped ensure the consistency and comparability of the data across hospitals. Third, our study did not include data on polypharmacy, which refers to the concurrent use of multiple medications. Polypharmacy is a common phenomenon among patients with T2DM, particularly those with multiple comorbidities, and has been shown to significantly influence healthcare costs. The absence of this variable may limit our ability to fully capture the drivers of medical expenditures, as polypharmacy can lead to increased costs related to drug acquisition, medication management, and potential adverse drug events. Lastly, only the in-patient medical expenditure of patients was described, while the medical services provided outside of these centers were not taken into account; hence, the medical expenditure may have been underestimated. In the future, studies with larger sample sizes and more comprehensive independent variables should be carried out to improve the accuracy of the estimates.

## 5 Conclusion

In conclusion, this study identified the factors affecting the medical expenditure of patients with T2DM. The results of this study can provide a reference for the medical security department to formulate a reasonable compensation plan and for medical institutions to optimize treatment plans, ultimately reducing the financial burden of patients and the pressure of medical insurance funds.

## Data Availability

The raw data supporting the conclusions of this article will be made available by the authors, without undue reservation.
