# Supplementary material for: In-patient expenditure between 2011 and 2021 for patients with type 2 diabetes mellitus: a hospital-based multicenter retrospective study in southwest China
Source: Front Public Health. 2025 Mar 10;13:1559424. doi: 10.3389/fpubh.2025.1559424 (PMC11931030; doi:10.3389/fpubh.2025.1559424)
Supplement: Supplementary file 1 [file Data_Sheet_1.docx]

**Supplementary information**

**Table S1. Socio-demographic and clinical-pathological characteristics of the 45237 selected patients, 2011-2021**

| Variable | Number of patients | | | | | | | | | | |  |
| --- | --- | --- | --- | --- | --- | --- | --- | --- | --- | --- | --- | --- |
|  | Total | 2011 | 2012 | 2013 | 2014 | 2015 | 2016 | 2017 | 2018 | 2019 | 2020 | 2021 |
| Age (years) |  |  |  |  |  |  |  |  |  |  |  |  |
| 18-44 | 9594 | 74 | 151 | 278 | 483 | 525 | 895 | 1309 | 1505 | 1745 | 1306 | 1323 |
| 45-64 | 17442 | 279 | 424 | 559 | 875 | 955 | 1549 | 2110 | 2664 | 3069 | 2440 | 2518 |
| 65-84 | 16987 | 211 | 396 | 525 | 833 | 888 | 1477 | 2103 | 2645 | 2977 | 2388 | 2544 |
| ≥85 | 1214 | 9 | 20 | 30 | 59 | 64 | 115 | 157 | 181 | 226 | 167 | 186 |
| Sex |  |  |  |  |  |  |  |  |  |  |  |  |
| female | 24980 | 302 | 534 | 815 | 1318 | 1441 | 2347 | 3208 | 3817 | 4510 | 3305 | 3383 |
| male | 20257 | 271 | 457 | 577 | 932 | 991 | 1689 | 2471 | 3178 | 3507 | 2996 | 3188 |
| Marital status |  |  |  |  |  |  |  |  |  |  |  |  |
| unmarried | 1011 | 4 | 14 | 37 | 30 | 81 | 127 | 123 | 118 | 173 | 136 | 168 |
| married | 41291 | 567 | 968 | 1342 | 2044 | 2118 | 3611 | 5161 | 6485 | 7331 | 5691 | 5973 |
| divorced/widowed | 2935 | 2 | 9 | 13 | 176 | 233 | 298 | 395 | 392 | 513 | 474 | 430 |
| Insurance type |  |  |  |  |  |  |  |  |  |  |  |  |
| full self-pay | 8554 | 0 | 2 | 96 | 122 | 271 | 1137 | 1430 | 1664 | 1593 | 1056 | 1183 |
| UEMI | 18375 | 2 | 93 | 615 | 991 | 1086 | 1585 | 2112 | 2806 | 3408 | 2828 | 2849 |
| URM | 14458 | 0 | 6 | 369 | 654 | 726 | 1030 | 1761 | 2261 | 2836 | 2363 | 2452 |
| NCMS | 451 | 2 | 5 | 2 | 50 | 43 | 62 | 105 | 65 | 30 | 38 | 49 |
| Other medical insurance | 3399 | 569 | 885 | 310 | 433 | 306 | 222 | 271 | 199 | 150 | 16 | 38 |
| Length of stay (days) |  |  |  |  |  |  |  |  |  |  |  |  |
| 2-4 | 8302 | 71 | 123 | 157 | 391 | 465 | 723 | 1084 | 1397 | 1554 | 1212 | 1125 |
| 5-7 | 13105 | 137 | 224 | 327 | 609 | 676 | 1197 | 1668 | 2112 | 2487 | 1799 | 1869 |
| 8-11 | 12164 | 127 | 297 | 388 | 594 | 622 | 1056 | 1523 | 1874 | 2135 | 1678 | 1870 |
| ≥12 | 11666 | 238 | 347 | 520 | 656 | 669 | 1060 | 1404 | 1612 | 1841 | 1612 | 1707 |
| Number of clinical visits |  |  |  |  |  |  |  |  |  |  |  |  |
| 1 | 26621 | 364 | 509 | 666 | 1106 | 1228 | 2017 | 3149 | 4017 | 4950 | 4078 | 4537 |
| 2 | 8981 | 75 | 151 | 270 | 450 | 498 | 818 | 1151 | 1523 | 1676 | 1206 | 1163 |
| 3 | 3917 | 38 | 94 | 144 | 226 | 266 | 445 | 525 | 670 | 638 | 467 | 404 |
| ≥4 | 5718 | 96 | 237 | 312 | 468 | 440 | 756 | 854 | 785 | 753 | 550 | 467 |
| Number of comorbidities |  |  |  |  |  |  |  |  |  |  |  |  |
| 0 | 23414 | 551 | 818 | 1204 | 1412 | 1552 | 2499 | 3040 | 3410 | 3791 | 2782 | 2355 |
| 1 | 8958 | 20 | 70 | 94 | 393 | 393 | 639 | 1038 | 1484 | 1727 | 1426 | 1674 |
| 2 | 6769 | 1 | 57 | 52 | 273 | 277 | 467 | 799 | 1131 | 1331 | 1085 | 1296 |
| 3 | 3715 | 1 | 31 | 23 | 108 | 129 | 251 | 485 | 596 | 716 | 621 | 754 |
| ≥4 | 2381 | 0 | 15 | 19 | 64 | 81 | 180 | 317 | 374 | 452 | 387 | 492 |
| History of disease |  |  |  |  |  |  |  |  |  |  |  |  |
| no | 8580 | 57 | 96 | 166 | 339 | 393 | 777 | 1094 | 1363 | 1572 | 1242 | 1481 |
| yes | 36657 | 516 | 895 | 1226 | 1911 | 2039 | 3259 | 4585 | 5632 | 6445 | 5059 | 5090 |
| History of surgery |  |  |  |  |  |  |  |  |  |  |  |  |
| no | 24870 | 304 | 523 | 726 | 1181 | 1331 | 2197 | 3147 | 3814 | 4436 | 3495 | 3716 |
| yes | 20367 | 269 | 468 | 666 | 1069 | 1101 | 1839 | 2532 | 3181 | 3581 | 2806 | 2855 |
| Smoking history |  |  |  |  |  |  |  |  |  |  |  |  |
| no | 32376 | 417 | 707 | 1010 | 1598 | 1794 | 2977 | 4147 | 5070 | 5676 | 4365 | 4615 |
| yes | 12861 | 156 | 284 | 382 | 652 | 638 | 1059 | 1532 | 1925 | 2341 | 1936 | 1956 |
| Drinking history |  |  |  |  |  |  |  |  |  |  |  |  |
| no | 33995 | 431 | 732 | 1019 | 1692 | 1852 | 3134 | 4334 | 5269 | 5986 | 4625 | 4921 |
| yes | 11242 | 142 | 259 | 373 | 558 | 580 | 902 | 1345 | 1726 | 2031 | 1676 | 1650 |
| ACCI score |  |  |  |  |  |  |  |  |  |  |  |  |
| 0-1 | 7553 | 55 | 94 | 300 | 434 | 501 | 710 | 1020 | 1116 | 1380 | 1031 | 912 |
| 2-3 | 10546 | 272 | 346 | 434 | 651 | 677 | 983 | 1240 | 1534 | 1740 | 1400 | 1269 |
| 4-5 | 13831 | 211 | 414 | 390 | 681 | 735 | 1334 | 1720 | 2140 | 2350 | 1864 | 1992 |
| ≥6 | 13307 | 35 | 137 | 268 | 484 | 519 | 1009 | 1699 | 2205 | 2547 | 2006 | 2398 |

**Table S2. The patient distribution across various comorbidity combinations, accompanied by their respective medical expenditures**

| Combination of Comorbidities | Number of Patients | Average Medical Expenditure |
| --- | --- | --- |
| pulmonary infection+hypertension | 202 | 24360 |
| CHD+HF+hypertension | 236 | 22029 |
| hyperlipidemia+CHD+hypertension | 201 | 22016 |
| kidney disease+CHD+HF+hypertension | 142 | 21690 |
| kidney disease+CHD+hypertension | 296 | 19560 |
| kidney disease+cancer+hypertension | 127 | 19315 |
| kidney disease+cancer | 130 | 18119 |
| cancer+hypertension | 302 | 18053 |
| osteoporosis+hypertension | 211 | 17777 |
| CHD+hypertension | 908 | 16975 |
| CHD+CI+hypertension | 155 | 16714 |
| kidney disease+CI+hypertension | 238 | 16528 |
| CI+hypertension | 869 | 14692 |
| hyperlipidemia+CI+hypertension | 213 | 13946 |
| HF+hypertension | 131 | 13543 |
| COPD+hypertension | 145 | 13366 |
| hyperlipidemia+hypertension | 923 | 13085 |
| kidney disease+hypertension | 1211 | 12921 |
| hyperlipidemia+kidney disease+hypertension | 447 | 12333 |
| hyperlipidemia+kidney disease | 371 | 11096 |

*HF:heart failure; CHD: coronary heart disease; COPD: chronic obstructive pulmonary disease; CI: cerebral infarction.*

**Figure S1. Patient flow diagram**

**
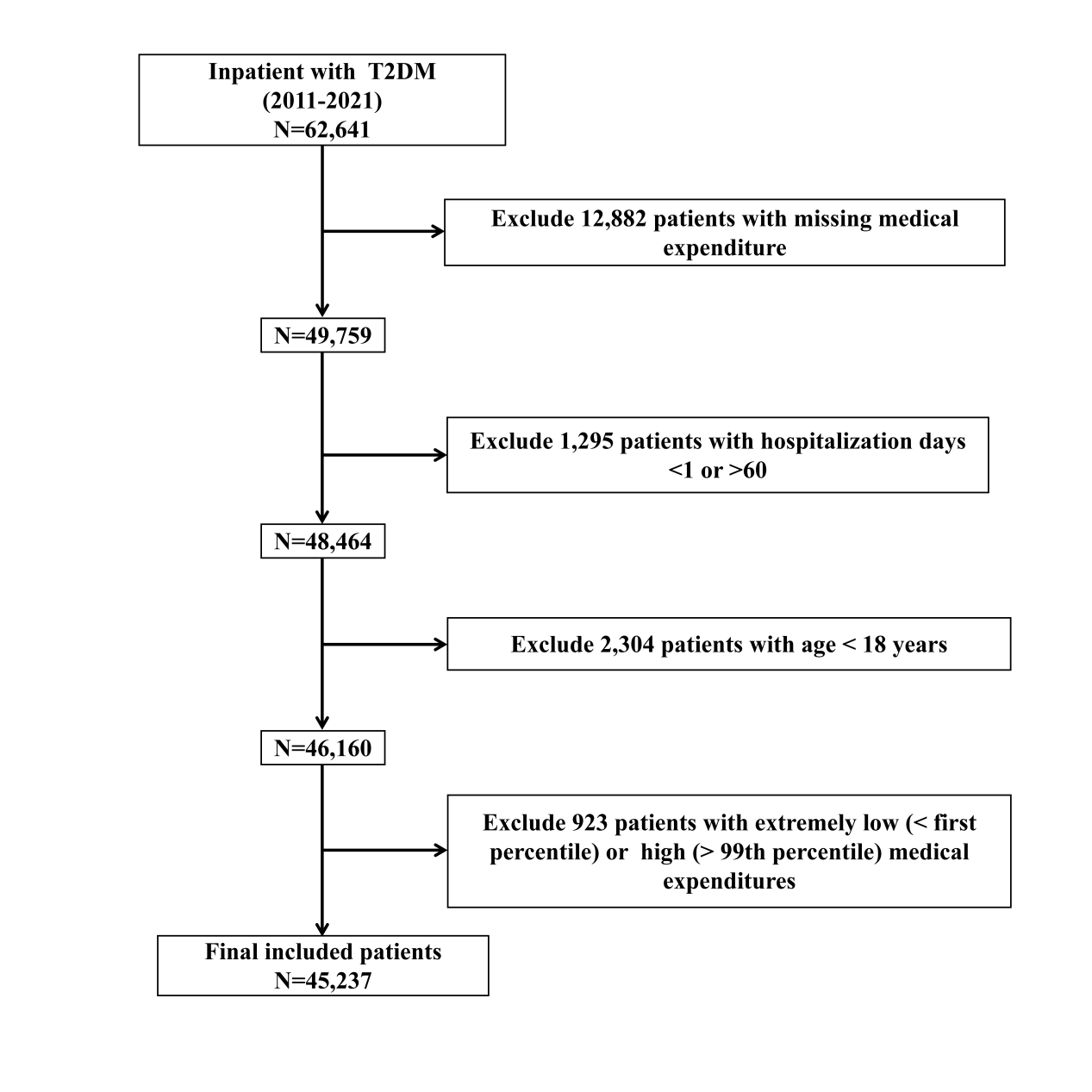
**

**Figure S2.** **Time trend of medical expenditure stratified by history of disease**

**
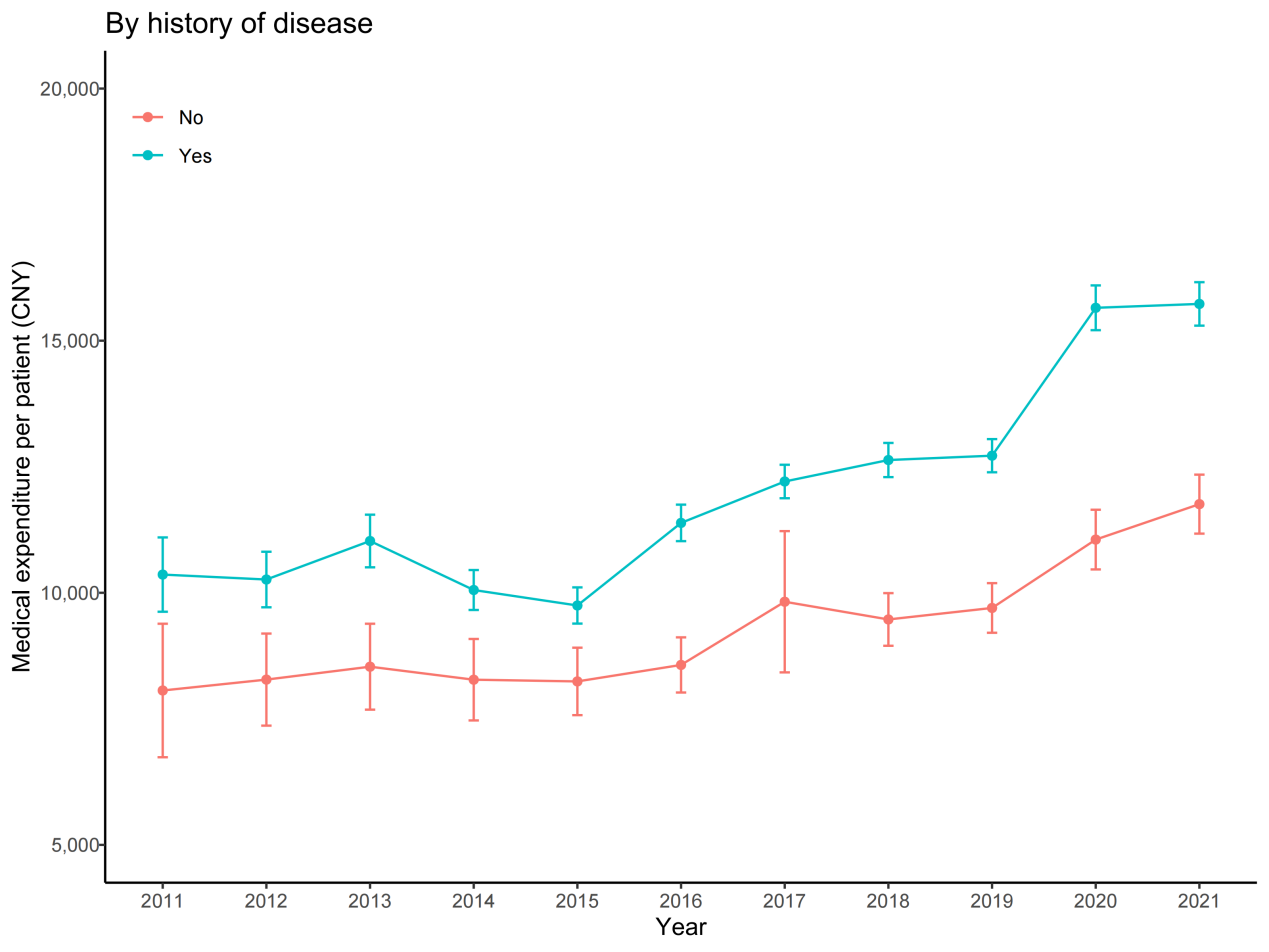
**

**Figure S3. Time trend of medical expenditure stratified by history of surgery**

**
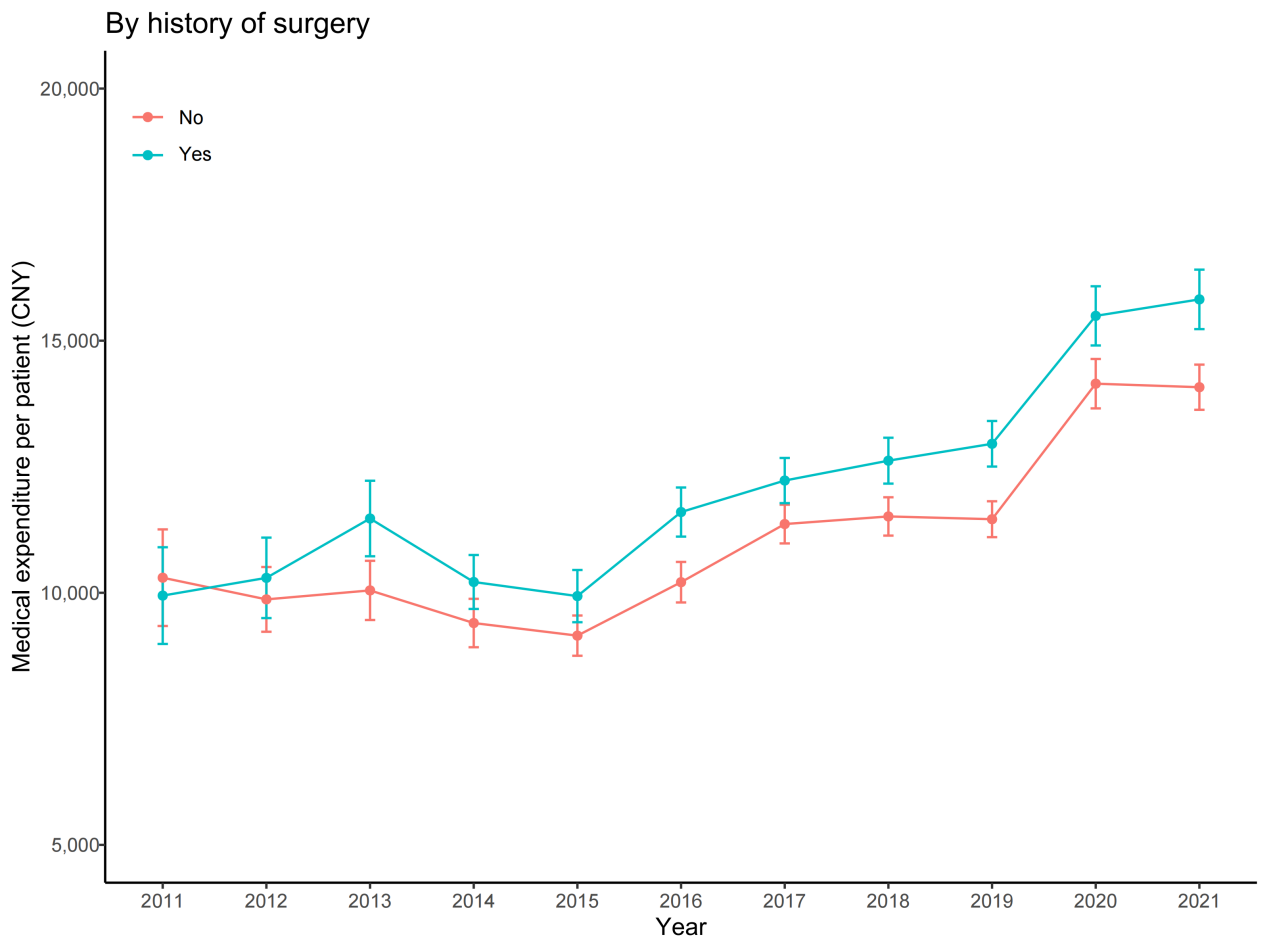
**

**Figure S4. Time trend of medical expenditure stratified by smoking history**

**
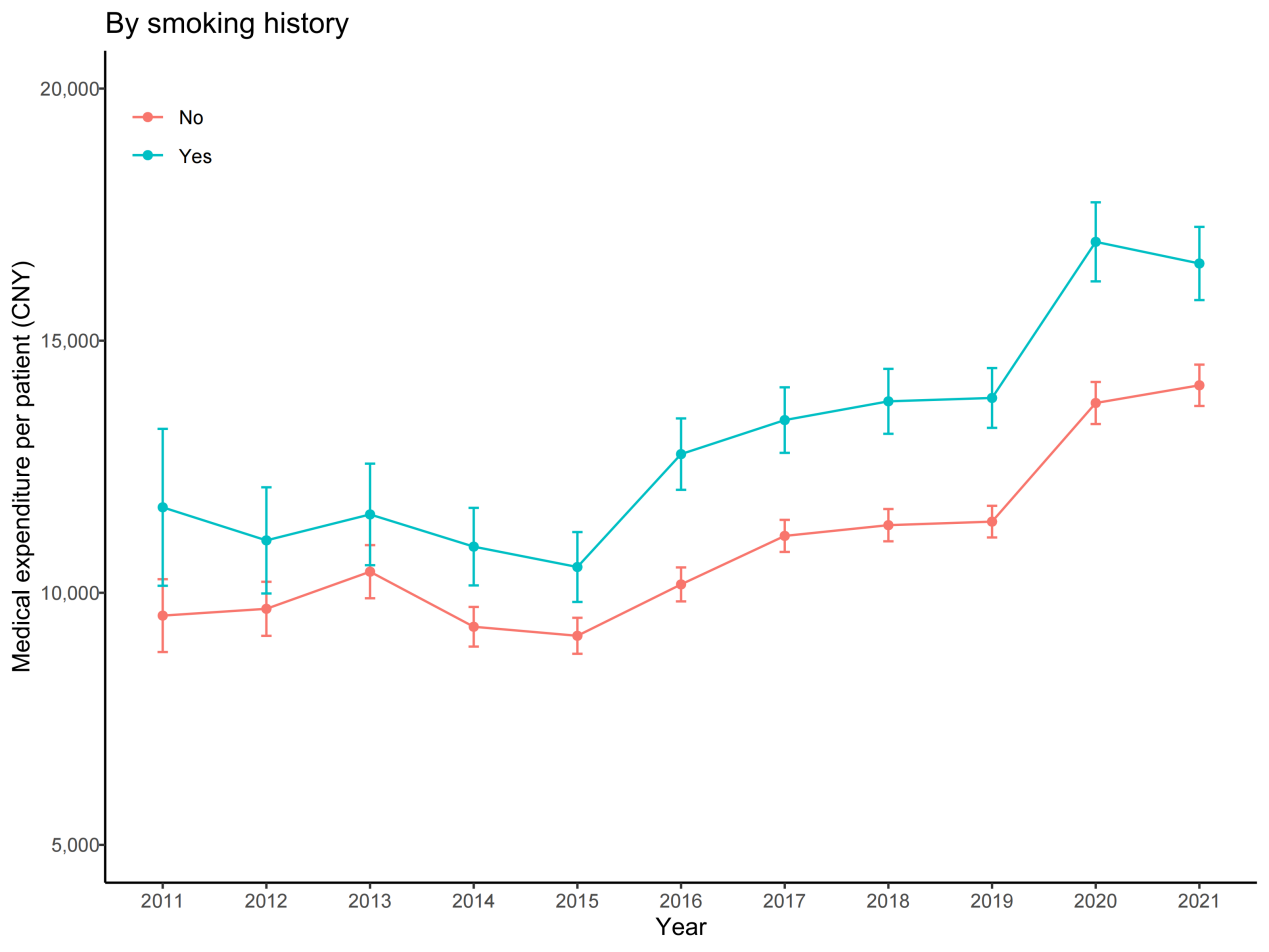
**
